# The involvement of beta-1,4-galactosyltransferase and N-acetylglucosamine residues in fertilization has been lost in the horse

**DOI:** 10.1186/1477-7827-6-51

**Published:** 2008-11-14

**Authors:** Sylvie Mugnier, Stéphane Boittin, Cécile Douet, Philippe Monget, Michèle Magistrini, Ghylène Goudet

**Affiliations:** 1 INRA, UMR85 Physiologie de la Reproduction et des Comportements, CNRS, Haras Nationaux, IFR 135, Université de Tours, F-37380 Nouzilly, France

## Abstract

**Background:**

In human and rodents, sperm-zona pellucida binding is mediated by a sperm surface Galactosyltransferase that recognizes N-Acetylglucosamine residues on a glycoprotein ZPC. In large domestic mammals, the role of these molecules remains unclear: in bovine, they are involved in sperm-zona pellucida binding, whereas in porcine, they are not necessary. Our aim was to clarify the role of Galactosyltransferase and N-Acetylglucosamine residues in sperm-zona pellucida binding in ungulates. For this purpose, we analyzed the mechanism of sperm-zona pellucida interaction in a third ungulate: the horse, since the Galactosyltransferase and N-Acetylglucosamine residues have been localized on equine gametes.

**Methods:**

We masked the Galactosyltransferase and N-Acetylglucosamine residues before the co-incubation of gametes. Galactosyltransferase was masked either with an anti-Galactosyltransferase antibody or with the enzyme substrate, UDP Galactose. N-Acetylglucosamine residues were masked either with a purified Galactosyltransferase or with an anti-ZPC antibody.

**Results and discussion:**

The number of spermatozoa bound to the zona pellucida did not decrease after the masking of Galactosyltransferase or N-Acetylglucosamine. So, these two molecules may not be necessary in the mechanism of in vitro sperm-zona pellucida interaction in the horse.

**Conclusion:**

The involvement of Galactosyltransferase and N-Acetylglucosamine residues in sperm-zona pellucida binding may have been lost during evolution in some ungulates, such as porcine and equine species.

## Background

The enzyme Beta-1,4-galactosyltransferase I (GalTase) was one of the first molecules involved in sperm-egg interaction that was studied [1,2]. GalTase was originally characterized for its role in oligosaccharide synthesis in the Golgi complex. At this location, GalTase adds galactose from uridine diphosphate galactose (UDP-Galactose) to N-acetylglucosamine (GlcNAc) residues on growing glycoprotein chains. GalTase was localized to the surface of spermatozoa as a plasma membrane protein [3]. It binds to terminal GlcNAc residues on O-linked oligosaccharides of ZPC [4,3].

GalTase was identified and localized in acrosome region in the plasma membrane of spermatozoa from human [5], rodents (mouse, rat, guinea pig), rabbit and ungulates (bull, boar, stallion) [6]. In human, mouse, and hamster,*in vitro*, when the GalTase or GlcNAc are masked, the number of spermatozoa bound to the zona pellucida decreases [7,1,2,8]. Thus, in these species, GalTase and GlcNAc are involved in the mechanism of *in vitro *sperm-zona pellucida binding. In ungulates, the role of GalTase and GlcNAc remains unclear. In bovine,*in vitro *GalTase masking inhibits the binding of spermatozoa to the zona pellucida [9]. On the contrary, in porcine species, Rebeiz and Miller [10] showed that masking of GalTase and GlcNAc did not disturb the binding of spermatozoa to the zona pellucida. So, the involvement of GalTase and GlcNAc in sperm-zona pellucida binding in ungulates has to be clarified.

In another ungulate, the horse, few studies were performed to identify the molecules that play a role in sperm-egg binding. GalTase was localized on the equine sperm head [6] and GalTase activity was mostly confined to the plasma membrane of equine spermatozoa [11]. GlcNAc residues were also observed on the equine zona pellucida and co-localized with the glycoprotein ZPC [12]. The GalTase on the sperm head and GlcNAc residues on the ZPC glycoprotein could bind during equine sperm-zona pellucida interaction. However, in the horse, no data are available about the role of these molecules in sperm-zona pellucida binding.

Our aim was to study the role of GalTase and GlcNAc during *in vitro *sperm-zona pellucida interaction in equine, in order to clarify the role of these molecules in fertilization in ungulates.

## Methods

Chemical products were purchased from Sigma (Saint-Quentin Fallavier, France) unless otherwise specified.

### Equine oocytes collection and maturation

Equine ovaries collected from a local slaughterhouse were transported at 30–37°C to the laboratory in 0.9% (w/v) NaCl diluted in H_2_O. Cumulus-oocyte-complexes (COCs) were aspirated from follicles using a 18.5 gauge needle at 50 mm Hg vacuum pressure before and after ovarian slicing.

*In vitro *maturation was performed in 500 μl of tissue culture medium 199 (TCM 199) supplemented with 50 ng ml^-1 ^Epidermal Growth Factor (EGF) [13] and 20% (v/v) Fetal Calf Serum (FCS). Maturation took place in a humidified atmosphere of 5% CO_2 _in air at 38.5°C for 30 hours. After *in vitro *culture, COCs were stripped of their cumulus cells with small glass pipettes in 500 μl Dulbecco's phosphate buffered saline solution (DPBS, Dulbecco A, Paris, France). The denuded oocytes were incubated in the IVF medium (see below).

### Preparation of semen

In each experiment, we first tested fresh semen, then frozen semen.

#### Preparation of fresh semen

Fresh equine semen was collected from a Welsh pony stallion from our experimental stud using an artificial vagina. It was prepared according to Palmer et al. [14], because these conditions allow the best IVF rate when using fresh semen. Briefly, immediately after collection, sperm was filtered and diluted to 25 × 10^6 ^spermatozoa ml^-1 ^in Hank's solution supplemented with 1% (w/v) BSA and 20 mmol l^-1 ^Hepes at pH 7.1 (HHBSA) [15]. It was preincubated at 37°C for 30 minutes in anaerobic conditions. Spermatozoa were then incubated with 6 μmol l^-1 ^of calcium ionophore A23187 (free acid) at 37°C for 5 minutes [16]. Spermatozoa were centrifuged for 3 minutes at 500 × g. The pellet was resuspended in HHBSA (25 × 10^6 ^spermatozoa ml^-1^). The motility was visually evaluated using an inverted epifluorescent microscope (Olympus, IMT-2, Paris, France).

#### Preparation of frozen semen

Two straws of semen (100 × 10^6 ^spermatozoa ml^-1^) from three Welsh pony stallions from our experimental stud were rapidly thawed during 30 secondes in a water bath at 37°C. Sperm was prepared according to Dell'Aquila et al. [17], because these conditions allow the best IVF rate when using frozen semen. Briefly, sperm cells were prepared using the swim-up procedure in Tyrode-lactate medium modified for sperm treatment (Sp-TALP). The chemical composition of Sp-TALP was Tyrode medium supplemented with 1 mmol l^-1 ^Pyruvate, 6 mg ml^-1 ^BSA (fatty acid free), 21 mmol l^-1 ^Lactate, 50 μg ml^-1 ^Gentamicine and 10 mmol l^-1 ^Hepes. Semen was layered (0.2 ml/tube) in a titled Falcon tube under 1 ml Sp-Talp and incubated at 38.5°C for 1 hour in 5% CO_2 _in air. The top (0.4 to 0.5 ml of medium) from each tube containing motile spermatozoa was removed, and the contents were pooled and centrifuged at 300 × g for 10 minutes. The supernatant was discarded and the pellet was resuspended in Sp-Talp for a total of 100 μl, then the concentration was calculated. The spermatozoa were diluted in Sp-TALP medium to 25 × 10^6 ^spermatozoa ml^-1^. The motility was visually evaluated with an inverted epifluorescent microscope (Olympus, IMT-2).

### IVF media

For fresh semen, the IVF medium was Synthetic Oviductal Fluid (SOF, for details of chemical composition, refer to Takahashi and First, [18] supplemented with 15% (v/v) FCS and 3.2 μg ml^-1 ^Gentamicine.

For frozen semen, the IVF medium was Tyrode-lactate medium modified for IVF treatment (Fert-TALP) as follow: Tyrode medium (100 mmol l^-1 ^NaCl, 3.1 mmol l^-1 ^KCl, 0.3 mmol l^-1 ^NaH_2_PO_4_.2H_2_O, 2.1 mmol l^-1 ^CaCl_2_, 0.4 mmol l^-1 ^MgCl_2_.6H_2_O, 10 mg ml^-1 ^Red Phenol, 25 mM NaHCO_3_) supplemented with 1 mmol l^-1 ^Pyruvate, 6 mg ml^-1 ^fatty acid free Bovine Serum Albumine (BSA), 21 mmol l^-1 ^Lactate, 50 μg ml^-1 ^Gentamicine, 1 μg ml^-1 ^Heparine [17].

### Experiment 1: GalTase masking with anti-GalTase antibodies

#### Assessment of the fixation of the anti-GalTase antibodies on spermatozoa

After spermatozoa preparation as previously described, diluted fresh or frozen spermatozoa were dried on slides at 37°C for 2 hours and fixed in ethanol/glacial acetic acid (95/5 v/v) for 10 minutes at -20°C in a humidified chamber. After fixation, each slide was dried at 37°C, covered with PBS-BSA solution (1% (w/v) fatty acid free BSA diluted in DPBS) for 1 hour and then, covered with anti-GalTase antibodies (Rabbit antiserum raised against bacterially expressed recombinant murine Beta-1,4-Galactosyltransferase I kindly donated by Dr. Barry Shur; [1]) or preimmune serum diluted 1/100 (as described by Larson and Miller [6]) in PBS-BSA solution for 2 hours.

Slides were washed with 1 ml PBS-BSA solution and covered with Fluoprobes 488-conjugated goat anti-rabbit antibodies (Goat anti-rabbit IgG F(AB')^2^, Interchim, Montluçon, France) diluted 1/100 in PBS-BSA solution for 2 hours in a covered humidified chamber to reduce the light exposure. Each slide was washed with 1 ml PBS-BSA solution and covered with Moviol V4-88 (133 mg ml^-1^, Hoechst, Frankfort, Germany) and finally with a coverslip. Slides were kept in darkness at 4°C till examination using an inverted epifluorescent microscope (Olympus, IMT-2) at magnification ×400.

#### GalTase masking with anti-GalTase antibodies

After spermatozoa preparation as previously described, fresh or frozen spermatozoa were incubated with anti-GalTase antibodies or preimmune serum diluted 1/100 in Sp-TALP (frozen semen) or HHBSA (fresh semen) or no additive. The spermatozoa were incubated for 30 minutes at 38.5°C in a humidified atmosphere of 5% CO_2 _in air.

In parallel, the *in vitro *matured oocytes (10 to 20 per well) were incubated with anti-GalTase antibodies or preimmune serum diluted 1/100 in the IVF medium (Fert-TALP or SOF) or no additive

### Experiment 2: GalTase masking with UDP Galactose

After spermatozoa preparation as previously described, diluted fresh or frozen spermatozoa were incubated in HHBSA or sp-TALP with 10 mmol l^-1 ^UDP Galactose (substrate of GalTase) or 10 mmol l^-1 ^UDP Glucose (sugar which is not a substrate of GalTase) or no additive at 38.5°C in a humidified atmosphere of 5% CO_2 _in air for 10 minutes.

In parallel, the *in vitro *matured oocytes (10 to 20 per well) were incubated in 500 μl IVF medium (Fert-TALP or SOF) with 10 mmol l^-1 ^UDP Galactose or 10 mmol l^-1 ^UDP Glucose or no additive for 10 minutes.

### Experiment 3: GlcNAc masking with purified GalTase

#### Assessment of the GalTase fixation on the zona pellucida

After 30 hours of maturation, oocytes were incubated with 500 μg ml^-1 ^GalTase (Beta-1,4-galactosyltransferase I human, Sigma, Biochemika, Fluka, Switzerland) or no additive in Fert-TALP or SOF medium for 1 hour at 38.5°C in humidified atmosphere of 5% CO_2_. The oocytes were fixed in 500 μl paraformaldehyde 2% in DPBS for 20 minutes at 37°C. After fixation, oocytes were washed with DPBS and incubated in PBS-BSA solution (5% (w/v) BSA in DPBS) for 1 hour at room temperature. The oocytes were incubated with anti-GalTase antibodies or preimmune serum diluted 1/50 in PBS-BSA solution for 2 hours at room temperature. After incubation, oocytes were washed with PBS-BSA solution and incubated in Fluoprobes 488-conjugated goat anti-rabbit antibodies diluted 1/100 in PBS-BSA for 2 hours in darkness at room temperature. Oocytes were washed with PBS-BSA solution, layed on a slide and covered with Moviol V4-88 and then, with a coverslip. Oocytes were kept in darkness at 4°C until examination using an inverted epifluorescent microscope (Olympus, IMT-2) at magnification ×400.

#### GlcNAc masking with purified GalTase

After 30 hours of maturation, oocytes (10 to 20 per well) were incubated in the IVF medium (Fert-TALP or SOF) with 500 μg ml^-1 ^GalTase or no additive for 1 hour at 38.5°C in humidified atmosphere of 5% CO_2 _in air. They were then transferred in IVF medium (Fert-TALP or SOF).

### Experiment 4: ZPC masking with anti-ZPC antibodies

After 30 hours of maturation, oocytes (10 to 20 per well) were washed in PBS-BSA (2% (w/v) fatty acid free BSA in DPBS), and then incubated with anti-ZPC antibodies (chicken anti-ZPC antibodies against porcine ZPC diluted 1/100 in PBS-BSA; kindly donated by Dr Sabine Kölle; [19]) or no additive for 1 hour at 38.5°C in humidified atmosphere of 5% CO_2 _in air.

The oocytes were then transferred in IVF medium (Fert-TALP or SOF).

### Gametes co-incubation and assessment of sperm-zona pellucida binding

After GalTase, GlcNAc or ZPC masking, the spermatozoa (final concentration of 5 × 10^5 ^cells ml^-1^) and the oocytes (10 to 20 per well) with similar treatments were co-incubated in 500 μl IVF medium for 20 minutes at 38.5°C in a humidified atmosphere of 5% CO_2 _in air.

After gametes co-incubation, oocytes were washed four times in DPBS in order to remove any unbound spermatozoa on the zona pellucida. Using an inverted microscope (Olympus IMT-2), the spermatozoa bound to the zona pellucida were counted in all focal plans at magnification ×400 by a blinded observer.

### Statistical analysis

For each experiment, due to the limited number of oocytes available on a single day, two to three replicates were performed. The mean and SEM of the number of spermatozoa bound to the zona pellucida per oocyte were calculated in each group of oocytes (controls and masking). Depending on the experiment, we compared the mean number of spermatozoa between groups (3 groups in experiments 1 and 2, 2 groups in experiment 3 and 4). When a significant effect between groups was observed then we compared the groups in pairs. Statistical difference between group means was determined using an analysis of variance (ANOVA). The alpha level was 5% and P-values < 0.05 were considered significant.

## Results

### Experiment 1: GalTase masking with anti-GalTase antibodies

#### Assessment of the fixation of the anti-GalTase antibodies on spermatozoa

**Figure 1:**
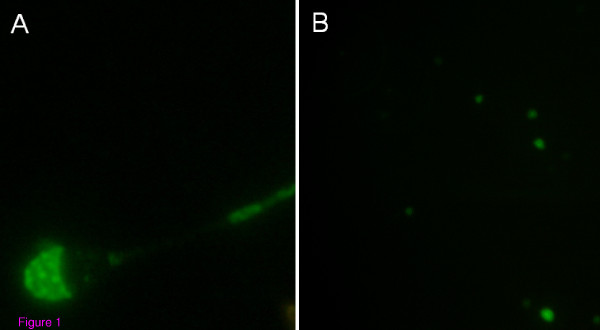
**Spermatozoa incubated with anti-GalTase antibodies (A) or with preimmune serum (B).** (Observation with an epifluorescent microscope at 400 × magnification).

In order to ascertain the binding of anti-GalTase antibodies on the spermatozoa, equine spermatozoa were incubated with anti-GalTase antibodies or preimmune serum. On the spermatozoa incubated with antibodies, staining was intense on the acrosomal region (figure 1A). The spermatozoa incubated with preimmune serum did not exhibit any staining (figure 1B). Thus, anti-GalTase antibodies are able to bind to the plasma membrane in the acrosomal region of spermatozoa.

#### GalTase masking with anti-GalTase antibodies

Table 1 shows that, when using fresh semen, the number of spermatozoa bound to the zona pellucida was not different between the three groups : anti-GalTase antibody, preimmune serum or no additive (P > 0.05).

**Table 1:** Number of fresh or frozen spermatozoa bound to the zona pellucida per oocyte after incubation of spermatozoa with anti-GalTase antibodies, preimmune serum or without any additive.

Groups		Anti-Galactosyltransferase antibodies	Preimmune serum	No additive
Fresh semen	No. Oocytes	31	28	28
	Spermatozoa/oocyte (mean ± SEM, n = 3)	99 ± 10	97 ± 9	112 ± 9
Frozen semen	No. Oocytes	37	37	36
	Spermatozoa/oocyte (mean ± SEM, n = 2)	10 ± 1^a^	19 ± 2^b^	7 ± 1^a^

a, b: statistical difference between the columns in the same line (for fresh semen F = 0.72 and p = 0.49, for frozen semen F = 19.78 and p < 0.001)

n: number of replicates

When using frozen semen, fewer spermatozoa were fixed on the zona pellucida in the presence of anti-Galtase antibodies or no additive than in the presence of preimmune serum (P < 0.001; Table 1). However, the number of spermatozoa bound to the zona pellucida was not different between the oocytes incubated with the antibodies and thoses incubated without any additive (P > 0.05; Table 1).

### Experiment 2: GalTase masking with UDP Galactose

The number of spermatozoa bound to the zona pellucida was not different after incubation with UDP Galactose or UDP Glucose or without any additive, when using fresh or frozen semen (P > 0.05; Table 2).

**Table 2:** Number of fresh or frozen spermatozoa bound to the zona pellucida of oocytes incubated with UDP Galactose, UDP Glucose or without any additive.

Groups		UDP Galactose	UDP Glucose	No additive
Fresh semen	No. Oocytes	35	34	33
	Spermatozoa/oocyte (mean ± SEM, n = 3)	42 ± 6	47 ± 7	64 ± 11
Frozen semen	No. Oocytes	37	38	38
	Spermatozoa/oocyte (mean ± SEM, n = 2)	29 ± 3	30 ± 2	28 ± 2

No statistical difference was observed between the columns in the same line (for fresh semen F = 1.97 and p = 0.14, for frozen semen F = 0.17 and p = 0.84)

n: number of replicates

### Experiment 3: GlcNAc masking with purified GalTase

#### Assessment of the GalTase fixation on the zona pellucida

**Figure 2:**
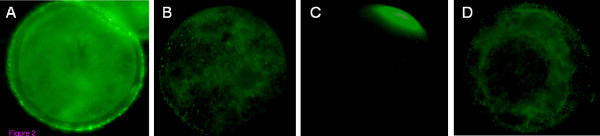
**Equine oocytes incubated with GalTase and with anti-GalTase antibodies (A) or preimmune serum (B); equine oocytes incubated without GalTase and with anti-GalTase antibodies (C) or preimmune serum (D).** (Observation with an epifluorescent microscope at 400 × magnification).

In order to ascertain the binding of GalTase on the zona pellucida, equine oocytes were incubated with GalTase or no additive and then, with anti-GalTase antibodies or preimmune serum. On the oocytes incubated with GalTase and anti-GalTase antibodies, the staining was intense on the zona pellucida (figure 2A). For the other conditions, no staining was observed on the oocytes (figure 2B, C, D). Thus, GalTase binds to the zona pellucida of oocytes.

#### GlcNAc masking with purified GalTase

The number of spermatozoa bound to the zona pellucida was not different after incubation with Galactosyltransferase or without any additive using fresh or frozen semen (P > 0.05; Table 3).

**Table 3:** Number of fresh or frozen spermatozoa bound to the zona pellucida per oocyte incubated with or without purified GalTase.

Groups		Galactosyltransferase	No additive
Fresh semen	No. Oocytes	43	42
	Spermatozoa/oocyte (mean ± SEM, n = 3)	99 ± 6	104 ± 12
Frozen semen	No. Oocytes	40	40
	Spermatozoa/oocyte (mean ± SEM, n = 2)	89 ± 5	91 ± 7

No statistical difference was observed between the columns in the same line (for fresh semen F = 0.14 and p = 0.70, for frozen semen F = 0.06 and p = 0.80)

n: number of replicates

### Experiment 4: ZPC masking with anti-ZPC antibodies

Table 4 shows that the number of spermatozoa bound to the zona pellucida was not different after incubation with anti-ZPC antibody or without any additive, when using fresh or frozen semen (P > 0.05; Table 4).

**Table 4:** Number of fresh or frozen spermatozoa bound to the zona pellucida of oocytes incubated with or without anti-ZPC antibodies.

Groups		Anti-ZPC antibodies	No additive
Fresh semen	No. Oocytes	31	32
	Spermatozoa/oocyte (mean ± SEM, n = 2)	60 ± 6	58 ± 5
Frozen semen	No. Oocytes	39	39
	Spermatozoa/oocyte (mean ± SEM, n = 2)	71 ± 7	64 ± 8

No statistical difference was observed between the columns in the same line (for fresh semen F = 0.05 and p = 0.82, for frozen semen F = 0.44 and p = 0.51)

n: number of replicates

## Discussion

As GalTase and GlcNAc are involved in sperm-zona pellucida binding in bovine, but not in porcine, our aim was to clarify the role of GalTase and GlcNAc in another ungulate. Our hypothesis was to know if GalTase and GlcNAc are involved in sperm-zona pellucida interaction in the horse. Our results show that these molecules are not essential for *in vitro *equine sperm-zona pellucida binding.

In the large domestic mammals, such as bull, boar and stallion, GalTase was localized on the plasma membrane of periacrosomal region of the sperm head [6,11]. Our experiments confirmed the GalTase localization on equine sperm. In order to investigate the role of GalTase, we analyzed sperm- zona pellucida binding when masking GalTase. The GalTase masking was performed with UDP Galactose as previously described in the mouse and in the pig or with anti-murine GalTase antibodies as previously described in the mouse and in the bull [2,10,9]. The percentage of sequence identity between horse and murine GalTase is 88%. We checked that in our *in vitro *conditions, the anti-GalTase antibodies previously used by Larson and Miller [6] in the equine, Lopez et al. [2] and Tengowski et al. [9] were actually able to bind the plasma membrane of the equine spermatozoa. Our results showed that, in our *in vitro *conditions, when using fresh or frozen semen, the GalTase masking did not modify sperm- zona pellucida binding, suggesting that GalTase alone is not necessary for the binding of spermatozoa to the zona pellucida in the horse. Previous reports demonstrated that, in porcine, blocking GalTase did not affect sperm- zona pellucida binding [10]. Thus, GalTase may not be essential for sperm- zona pellucida binding in equine and porcine species. On the contrary, in bovine, during *in vitro *gametes co-incubation, GalTase plays a role in the sperm-zona pellucida binding: GalTase masking with anti-GalTase antibodies decreased the number of spermatozoa bound to the zona pellucida [9]. Thus, the involvement of GalTase in sperm-zona pellucida binding may be different between species within the ungulates.

In the horse, we showed previously that GlcNAc residues were present on the equine zona pellucida and co-localized with the glycoprotein ZPC [12]. These data suggest that GlcNAc may be linked to ZPC as observed in the mouse [3,4]. In order to investigate the role of GlcNAc in sperm- zona pellucida binding, we masked these residues with purified GalTase as previously described in the mouse [2]. In our *in vitro *conditions, we ascertained the binding of purified GalTase to the ZP. We also masked the ZPC protein with anti-ZPC antibodies, in order to decrease the accessibility of the GlcNAc residues. We ascertained previously the binding of anti-porcine ZPC antibodies on the equine zona pellucida [12]. Moreover, the percentage of sequence identity between horse and porcine ZPC is 76%. Our results showed that, in our *in vitro *conditions, when using fresh or frozen semen, the GlcNAc masking did not modify the binding of spermatozoa to the zona pellucida. Similar results were observed in the porcine species: removal of the GlcNAc residues by incubation of oocytes with N-Acetylglucosaminidase did not affect sperm- zona pellucida binding [10]. Thus, in horse and pig, GlcNAc residues are not essential for gametes interaction. On the contrary, in the mouse, incubation of oocytes with N-Acetylglucosaminidase decreased the number of spermatozoa bound to the zona pellucida [1]. In addition, the presence of purified GalTase produced a dose-dependant inhibition of sperm binding to the zona pellucida [2]. In human and hamster, the presence of GlcNAc before or during *in vitro *male and female gametes co-incubation, reduced the ability of spermatozoa to bind to the zona pellucida [7,8]. Thus, in human and rodents, GlcNAc participates in the *in vitro *sperm- zona pellucida binding, whereas in horse and pig, GlcNAc may not be essential.

**Figure 3:**
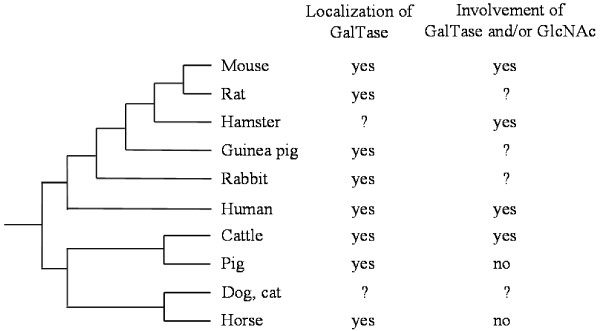
Simplified tree of life showing the localization of GalTase on the spermatozoa, or the involvement of GalTase and/or GlcNAc in sperm-zona pellucida binding in mammals.

In summary, GalTase and GlcNAc residues are involved in sperm-zona pellucida binding in human and rodents, as well as bovine. On the contrary, GalTase and GlcNAc residues are not essential in porcine and equine species. A schematic tree of life (according to [20]) showing the involvement of GalTase and/or GlcNAc in sperm-zona pellucida binding in mammals is presented in Figure 3. In human and rodents, the role of GalTase and GlcNAc in sperm-zona pellucida binding seems to be maintained during evolution. Among ungulates, the role of GalTase and GlcNAc may have been lost during evolution in pigs and horses, but not in cattle. To our knowledge, no data are available about the involvement of GalTase and GlcNAc in sperm-zona pellucida binding in dog and cat. If GalTase and GlcNAc residues are not essential in dog and cat, the involvement of these molecules would have been maintained only in cattle. Further studies are necessary to clarify this point. Whether GalTase gene is subjected to positive selection during evolution remains to be investigated. Of note, genes involved in reproduction and in sperm-egg interaction such as sperm-specific genes are under fast evolution [21].

In equine species, GalTase and GlcNAc are not essential for sperm-zona pellucida interaction, but other molecules could be involved. For example, in the equine spermatozoa, HSP-7 could play a potential role in sperm- zona pellucida interaction. This 14 KDa protein, isolated from stallion seminal plasma, belongs to the spermadhesin protein family, sharing 98% sequence identity with the porcine seminal plasma protein AWN-1 [22]. Like its boar homolog, HSP-7 is able to bind to the zona pellucida [23]. Some other molecules, localized on the plasma membrane of the spermatozoa in other mammals, could play a role in the equine species: SED1, fertilin β and peroxiredosin 5 identified in the porcine species, ADAM2-ADAM3 complex on the mouse spermatozoa, N-Acetylglucosaminidase in the human spermatozoa and Arylsulfatase A in the mouse or the pig [[24-27], [28] for review, [29] for review]. On the zona pellucida, several carbohydrate domains could be involved in sperm binding: O- and N-linked chains, the nonreducing terminal β-galactosyl residues and the alpha D mannose residues seem to participate in the mechanism of porcine sperm- zona pellucida binding [30-33]. Moreover, sperm binding could involve protein domains. In mouse, the sperm receptor on the zona pellucida would be the ZPC polypeptide because deglycosylated ZPC inhibited the sperm- zona pellucida binding [34]. In human, binding of sperm to zona proteins does not require the presence of glycan moieties [35]. Finally, sperm-zona pellucida binding may involve a multimeric complex incorporating several discrete molecular entities. For example, sperm from GalTase-null mouse is still able to fertilize the oocytes *in vivo*, though it is less able to undergo the acrosome reaction, penetrate the zona pellucida and fertilize the oocyte *in vitro *[36]. Mouse oocytes with lacking terminal GlcNAc residues are able to be fertilized *in vivo *[37]. Thus, *in vivo*, it is likely that compensatory processes enable sperm-ZP binding due to redundant gamete receptors. Finally, sperm binding could be dependent on a species specific supra-molecular structure of the zona matrix, zona pellucida proteins constituting a three-dimensional structure to which sperm would bind [38].

## Competing interests

The authors declare that they have no competing interests.

## Authors' contributions

SM, GG, MM and PM participated in the design of the study. SM, GG and PM wrote the manuscript. SM, SB, CD and GG performed gametes collection and preparation, and masking experiments. All authors read and approved the final manuscript.
